# Access to HIV/STI testing among male and female Venezuelan migrants in Peru: evidence from a nationwide survey

**DOI:** 10.1186/s12889-024-17655-2

**Published:** 2024-01-17

**Authors:** Ali Al-kassab-Córdova, Carolina Mendez-Guerra, Pamela Robles-Valcarcel, Guido Bendezu-Quispe, Vicente A. Benites-Zapata

**Affiliations:** 1 Escuela de Medicina, Universidad César Vallejo, Trujillo, Peru; 2 Facultad de Ciencias de la Salud, Universidad Peruana de Ciencias Aplicadas, Lima, Peru; 3 Universidad Privada Norbert Wiener, Lima, Peru; 4 Unidad de Investigación para la Generación y Síntesis de Evidencias en Salud, Universidad San Ignacio de Loyola, Lima, Peru

**Keywords:** Transients and migrants, Refugees, HIV testing, HIV, Sexually transmitted diseases, Peru, Venezuela, Latin America

## Abstract

**Background:**

Human immunodeficiency virus (HIV) infection and sexually transmitted infections (STIs) are major global public health issues. Migrants represent a vulnerable group that faces multiple barriers to access to healthcare services, including HIV/STI testing. This study aimed to assess the factors associated with access to HIV/STI testing in male and female Venezuelan migrants in Peru.

**Methods:**

This was a cross-sectional study involving secondary data analysis of the 2022 Venezuelan Population Residing in Peru Survey. The study was conducted in the eight most populated cities inhabited by Venezuelan migrants and refugees. For each city, the sampling design was probabilistic, stratified, and independent. The outcome variable was whether participants had access to HIV or other STI testing during their stay in Peru. Statistical analysis was stratified by sex owing to potential effect modification. Crude and adjusted prevalence ratios were calculated using generalized linear models *Poisson* family with *log* link function. Confidence intervals were calculated to 95%.

**Results:**

A total of 3,723 male and 3,984 female migrants were included. Access to HIV/STI testing among male and female migrants was 19.85% and 25.16%, respectively. Among male migrants, being LGBTI, health insured, and married or cohabiting were associated with increased access to HIV/STI testing. Among females, those aged 18–44 years, those who were married or cohabiting and were health insured, and those residing for more than 1 year in Peru were significantly more likely to have access to HIV/STI testing. Moreover, physical/mental disability and unemployed status were associated with a lower probability of HIV/STI testing in females.

**Conclusions:**

Only two in ten Venezuelan migrants and refugees in Peru were screened for HIV/STI, with fewer males than females. Sex-specific sociodemographic, health-related, and migration-related variables were independently associated with access to HIV/STI testing.

## Background

Human immunodeficiency virus (HIV) infection and sexually transmitted infections (STIs) continue to be major public health issues worldwide. Despite the decreasing trends in HIV/Acquired Immunodeficiency Syndrome (AIDS) incidence, disability-adjusted life years, and death over the past 15 years, the global prevalence of HIV infection and STIs has been increasing [1]. Over the past two decades, the incidence rates of trichomoniasis and genital herpes have remained stagnant, those of chlamydia and gonorrhea have decreased, and those of syphilis have increased [2]. Although the estimated cost for implementing the Global Strategy for STI in 117 low- and middle-income countries during 2016–2021 was 18.1 billion dollars [3], considerable disparities exist among countries regarding their spending on health and HIV/AIDS [4].

Early diagnosis of HIV/STI followed by prompt initiation of therapy is the cornerstone for HIV/STI control because delayed diagnosis and treatment discontinuation are associated with increased morbidity and mortality [5–7]. Specific vulnerable populations, including migrants, are at a greater risk of delayed diagnosis of HIV/STI and its harmful consequences. Migrants represent a disadvantaged group that faces multiple barriers impeding their access to healthcare services, exacerbating their vulnerability [8, 9]. Therefore, they are at a greater risk of delayed diagnosis of HIV/STI [10].

Peru has the second-highest number of Venezuelan migrants in the world. According to official data, the country hosts more than 1.5 million Venezuelan asylum-seekers, refugees, and migrants [11]. These migrants typically face several barriers that impede their access to healthcare services, including lack of insurance, financial constraints, and illegal migratory status [12]. Of note, the reported prevalence rates of individuals living with HIV in Venezuela and Peru were 0.5% [13] and 0.4% [14], respectively. Furthermore, owing to limited employment opportunities, some Venezuelan migrants resort to prostitution. This has led to the sexualization and stigmatization of Venezuelan migrants in Peru as they are “blamed” for importing HIV/STI [15]. In this context, the provision of sexual and reproductive health services to Venezuelan migrants is a key imperative. This study aimed to assess the factors associated with HIV/STI testing in male and female Venezuelan migrants in Peru.

## Methods

### Study design, population, sampling, sample size, and data source

This cross-sectional study was based on a secondary data analysis of the 2022 Venezuelan Population Residing in Peru Survey (ENPOVE-2022, from the Spanish acronym). The survey was conducted by the National Institute of Statistics and Informatics (INEI, from the Spanish acronym) from February to March 2022 in the following eight most populated cities inhabited by Venezuelan migrants and refugees: Lima and Callao, Arequipa, Chiclayo, Chimbote, Ica, Piura, Tumbes, and Trujillo. These provincial capitals have the highest number of dwellings with Venezuelan residents at the national level, accounting for 82.9% of the total. ENPOVE-2022 involved direct interviews of Venezuelan migrants with 138 questions, the responses to which were recorded on tablets. The questions were related to several aspects of the Venezuelan population in Peru, including housing, household and resident characteristics, migration status, health, education, employment, discrimination, gender, and victimization.

ENPOVE-2022 targeted the Venezuelan population residing in private and collective dwellings in urban areas. The sampling frame was generated using data from the National Labour Market Survey and the National Superintendence of Migration. The sampling units were Venezuelan-populated households, households within those households, and Venezuelan residents in households. For each city, the sampling design was probabilistic, stratified, and independent. Individuals aged over 12 years were directly interviewed, while the youngest members of the family were surveyed through the head of the household. A total of 3,680 Venezuelan households were included in the sample. Detailed information regarding the survey is available elsewhere [12]. In the present study, Venezuelan migrants and refugees living in Peru who were aged over 18 years (the Peruvian age of majority) and for whom complete information on the variables of interest was available were included.

### Outcome

The outcome variable was whether participants had access to HIV or other STI testing during their stay in Peru. Participants were asked, “Have you or your partner had access to testing for HIV and other STIs”? Affirmative responses were deemed as having the outcome, whereas negative responses were deemed as not having the outcome. Participants who answered “do not know” were excluded.

### Covariates

Sociodemographic variables included age (18–24, 25–34, 35–44, 45–54, 55–64, and 65 years or older), sexual orientation (heterosexual/LGBTI), educational level (no formal education or primary/secondary/higher), socioeconomic status (low/middle/high), employment status (employed/unemployed), marital status (married or cohabiting/others), and city of residence. Health-related variables included mental or physical disability (yes/no) and health insurance (insured/uninsured). Migration-related variables included migratory status (legal/illegal), perceived discrimination (yes/no), and time residing in Peru (0–6, 7–12, or more than 12 months).

### Statistical analysis

Given the complex survey design, statistical analysis was performed using the *svy* package in Stata 16.0 (Stata Corporation, College Station, TX, USA). Sex was evaluated as a potential effect modifier of the associations using a homogeneity test (the Mantel–Haenszel method). Most of the tested scenarios showed significant differences according to sex. Therefore, all analyses were stratified by sex. Descriptive analyses were performed using absolute and weighted relative frequencies. Differences in access to HIV/STI testing according to each category of the covariates were assessed using the chi-square test with Rao–Scott correction. Variables that were associated with a *p*-value < 0.2 in the bivariate analysis were included in the multivariable analysis. To assess the magnitude of the association through crude and adjusted prevalence ratios (aPR), generalized linear models of the *Poisson *family with *log *link function were performed. Confidence intervals (CI) were computed to a 95%, and *p*-values < 0.05 were considered indicative of statistical significance.

## Results

### Participant characteristics

A total of 3,723 male and 3,984 female Venezuelan migrants living in Peru were included. The characteristics of the study population are summarized in Table 1. Most respondents were 25–34 years old (male, 43.7%; female, 38.2%), heterosexual (male, 97.9%; female, 98.3%), and had low socioeconomic status (male, 38.2%; female, 40.7%). Additionally, the majority had no disability (male, 98.2%; female, 97.8%), were employed (male, 90.9%; female, 65.2%), and held a legal migratory status (male, 72.6% male; female, 68.3%). Almost two-thirds were married or living with a partner (male, 66.8%; female, 63.4%) and three-quarters resided in Peru for more than 1 year (male, 84.6%; female, 84.5%), mainly in Lima (male, 82.6%; female, 82.9%). Finally, most male respondents had secondary education (48.5%) and were uninsured (76.8%), whereas most female respondents had higher education (50.7%) and were covered by health insurance (75.0%). Access to HIV/STI testing was 19.85% (95% CI, 17.99–21.85) and 25.16% (95% CI, 23.07–27.37) in male and female respondents, respectively.

**Table 1:** Characteristics of the study population disaggregated by sex (*n* = 7,707)

Characteristics		Male		Female	
		Absolute frequency	Weighted proportion	Absolute frequency	Weighted proportion
		N	%	n	%
Age (years)					
	18–24	830	18.65	902	20.01
	25–34	1,705	43.67	1,608	38.17
	35–44	908	22.69	872	21.03
	45–54	396	9.67	525	11.69
	55–64	168	3.71	274	6.44
	65 or older	74	1.62	117	2.66
Sexual orientation					
	Heterosexual	3,640	97.89	3,913	98.25
	LGBTI	91	2.11	60	1.75
Education level					
	No formal education or primary	536	10.3	486	8.86
	Secondary	1,781	48.49	1,664	40.44
	Higher	1,420	41.21	1,840	50.7
Socioeconomic status					
	Lower	1,723	38.18	1,941	40.69
	Middle	1,506	37.04	1,529	36.3
	Higher	983	24.78	942	23.01
Mental or physical disability					
	Yes	69	1.79	84	2.16
	No	3,789	98.21	4,011	97.84
Employment status					
	Employed	3,379	90.93	2,557	65.22
	Unemployed	358	9.07	1,433	34.78
Health insurance					
	Uninsured	3,098	76.8	876	25.04
	Insured	760	23.2	3,219	74.96
Migratory status					
	Legal	2,521	72.61	2,522	68.33
	Illegal	1,337	27.39	1,573	31.67
Time residing in Peru (in months)					
	0–6	384	9.16	403	9.03
	7–12	265	6.24	293	6.48
	More than 12	3,209	84.6	3,399	84.49
Marital status					
	Married or living with a partner	2,749	66.78	2,734	63.37
	Other	1,332	33.22	1,564	36.63
Perceived discrimination					
	Yes	1,215	31.84	1,419	34.99
	No	2,522	68.16	2,571	65.01
City of residence					
	Arequipa	237	3.43	235	3.32
	Chiclayo	239	1.52	283	1.77
	Chimbote	290	1.63	294	1.52
	Ica	240	2.65	209	2.17
	Lima	2,211	82.59	2,344	82.89
	Piura	250	2.23	268	2.26
	Trujillo	464	4.87	486	4.98
	Tumbes	281	1.08	293	1.08

### Covariates and access to HIV/STI testing disaggregated by sex

Most covariates showed a significant association with access to HIV/STI testing in male respondents, except for socioeconomic status, mental or physical disability, employment status, and perceived discrimination. Those aged 25–34 years (22.7%), those identifying as LGBTI (49.5%), those with higher education (24.3%), without health insurance (26.9%), married or living with a partner (21.7%), with a legal migratory status (21.5%), and those living in Chiclayo (36.5%) had greater access to HIV/STI testing (Table 2).

**Table 2:** Characteristics of the study population with or without access to HIV/STI testing disaggregated by sex (*n* = 7,707)

Characteristics		HIV/STI testing					
		Male			Female		
		No (%)	Yes (%)	*p* value*	No (%)	Yes (%)	*p* value*
Age (years)				**< 0.001**			**< 0.001**
	18–24	85.07	14.93		71.52	28.48	
	25–34	77.29	22.71		68.1	31.9	
	35–44	78.5	21.5		77.18	22.82	
	45–54	82.96	17.04		85.27	14.73	
	55–64	89.23	10.77		92.78	7.23	
	65 or older	89.26	10.74		92.25	7.75	
Sexual orientation				**< 0.001**			0.414
	Heterosexual	80.81	19.19		74.8	25.2	
	LGBTI	50.46	49.54		81	19	
Education level				**<0.001**			0.102
	No formal education or primary	82.86	17.14		77.35	22.65	
	Secondary	83.38	16.62		76.62	23.38	
	Higher	75.66	24.34		72.99	27.01	
Socioeconomic status				0.814			0.212
	Lower	79.73	20.27		77.21	22.79	
	Middle	79.86	20.14		73.31	26.69	
	Higher	81.22	18.78		73.02	26.98	
Mental or physical disability				0.349			**<0.001**
	Yes	74.72	25.28		93.62	6.38	
	No	80.25	19.75		74.42	25.58	
Employment status				0.6571			**0.002**
	Employed	78.92	21.08		70.93	29.07	
	Unemployed	80.28	19.72		76.93	23.07	
Health insurance				**< 0.001**			**< 0.001**
	Uninsured	82.12	17.88		77.52	22.48	
	Insured	73.05	26.95		66.1	33.9	
Migratory status				**0.002**			0.658
	Legal	78.52	21.48		74.54	25.46	
	Illegal	84.53	15.47		75.47	24.53	
Time residing in Peru (in months)				0.072			**0.002**
	0–6	87.06	12.94		84.94	15.06	
	7–12	83.09	16.91		76.97	23.03	
	More than 12	79.34	20.66		73.75	26.25	
Marital status				**0.008**			**< 0.001**
	Married or living with a partner	78.34	21.66		68.99	31.01	
	Other	83.58	16.42		84.55	15.45	
Perceived discrimination				0.236			**0.001**
	Yes	78.64	21.36		70.5	29.5	
	No	80.86	19.14		77.17	22.83	
City of residence				**< 0.001**			**< 0.001**
	Arequipa	81.15	18.85		74.08	25.92	
	Chiclayo	63.47	36.53		63.97	36.03	
	Chimbote	69.88	30.12		54.96	45.04	
	Ica	81.86	18.14		69.5	30.5	
	Lima	80.85	19.15		75.75	24.25	
	Piura	74.14	25.86		73.57	26.43	
	Trujillo	81.16	18.84		76.38	23.62	
	Tumbes	60.6	39.4		55.27	44.73	

*Chi-squared test with Rao–Scott correction

LGBTI: lesbian, gay, bisexual, transgender and intersex

Note: Statistically significant values (*p* < 0.05) are indicated in bold

Several covariates were significantly associated with access to HIV/STI testing in female respondents, including sexual orientation, educational level, socioeconomic status, and migratory status. Those aged 18–24 years (28.5%), those with no mental or physical disability (25.6%), employed (29.1%), uninsured (22.5%), having perceived discrimination (29.5%), living in Peru for more than 1 year (26.3%), married or living with a partner (31.0%), and those residing in Chimbote (45.0%) had greater access to HIV/STI testing (Table 2).

### Factors associated with access to HIV/STI testing disaggregated by sex

Several covariates showed an independent association with access to HIV/STI testing among male migrants. LGBTI migrants had a 2.31-fold greater likelihood of accessing HIV/STI testing than heterosexual migrants (aPR = 2.31; 95% CI, 1.67–3.20). Individuals with health insurance were significantly more likely to have accessed HIV/STI testing compared to those not having health insurance (aPR = 1.35; 95% CI, 1.11–1.64). Regarding marital status, being married or living with a partner was associated with a 1.30-fold greater likelihood of HIV/STI testing than any other status (aPR = 1.30; 95% CI, 1.04–1.63). Venezuelan migrants residing in Chiclayo, Chimbote, Piura and Tumbes had a 2.09-fold (aPR = 2.09; 95% CI, 1.58–2.76), 1.81-fold (aPR = 1.81; 95% CI, 1.37–2.38), 1.50-fold (aPR = 1.50; 95% CI, 1.07–2.11), and 2.48-fold (aPR = 2.48; 95% CI 1.88–3.26) greater likelihood of access to HIV/STI testing, respectively, compared with their counterparts residing in Lima. Venezuelan migrants with secondary education were significantly less likely to have access to HIV/STI testing compared with their counterparts with high-level education (aPR = 0.79; 95% CI, 0.65–0.97) (Table 3).

**Table 3:** Factors associated with HIV/STI testing among male respondents (*n* = 3,723)

Characteristics		Crude^a^			Adjusted^b^		
		cPR^c^	95% CI^d^	*p*-value	aPR^e^	95% CI^d^	*p*-value
Age (years)							
	18–24	1.39	0.50–3.83	0.524	1.71	0.60–4.83	0.310
	25–34	2.11	0.78–5.73	0.141	2.10	0.75–5.81	0.153
	35–44	2.00	0.73–5.48	0.177	1.96	0.70–5.48	0.198
	45–54	1.58	0.56–4.43	0.378	1.57	0.55–4.47	0.395
	55–64	1.00	0.33–3.02	0.996	1.00	0.32–3.05	0.994
	65 or older	Ref.	Ref.	Ref.	Ref.	Ref.	Ref.
Sexual orientation							
	Heterosexual	Ref.	Ref.	Ref.	Ref.	Ref.	Ref.
	LGBTI	2.58	1.91–3.47	**< 0.001**	2.31	1.67–3.20	**< 0.001**
Education level							
	No formal education or primary	0.70	0.51–0.96	**0.029**	0.82	0.58–1.16	0.271
	Secondary	0.68	0.56–0.82	**< 0.001**	0.79	0.65–0.97	**0.031**
	Higher	Ref.	Ref.	Ref.	Ref.	Ref.	Ref.
Health insurance							
	Uninsured	Ref.	Ref.	Ref.	Ref.	Ref.	Ref.
	Insured	1.50	1.25–1.81	**< 0.001**	1.35	1.11–1.64	**0.003**
Migratory status							
	Legal	0.71	0.58–0.88	**0.002**	0.84	0.66–1.06	0.151
	Illegal	Ref.	Ref.	Ref.	Ref.	Ref.	Ref.
Time residing in Peru (in months)							
	0–6	Ref.	Ref.	Ref.	Ref.	Ref.	Ref.
	7–12	1.30	0.68–2.47	0.412	1.35	0.72–2.51	0.342
	More than 12	1.59	1.05–2.42	**0.027**	1.28	0.83–1.97	0.254
Marital status							
	Married or living with a partner	1.31	1.07–1.62	**0.009**	1.30	1.04–1.63	**0.019**
	Other	Ref.	Ref.	Ref.	Ref.	Ref.	Ref.
City of residence							
	Arequipa	0.98	0.67–1.44	0.937	0.95	0.68–1.35	0.812
	Chiclayo	1.90	1.47–2.47	**< 0.001**	2.09	1.58–2.76	**< 0.001**
	Chimbote	1.57	1.21–2.03	**0.001**	1.81	1.37–2.38	**< 0.001**
	Ica	0.94	0.66–1.35	0.764	1.10	0.78–1.55	0.577
	Lima	Ref.	Ref.	Ref.	Ref.	Ref.	Ref.
	Piura	1.35	0.95–1.91	0.093	1.50	1.07–2.11	**0.018**
	Trujillo	0.98	0.71–1.35	0.923	1.04	1.76–1.41	0.797
	Tumbes	2.05	1.57–2.68	**<0.001**	2.48	1.88–3.26	**< 0.001**

^a^ Poisson regression

^b^ Poisson regression adjusted per all model variables

^c^ cPR: crude prevalence ratio

^d^ 95% CI: 95% confidence interval

^e^ aPR: adjusted prevalence ratio

LGBTI: lesbian, gay, bisexual, transgender and intersex

Note: Statistically significant values (*p* < 0.05) are indicated in bold

Females aged 18–24, 25–34, and 35–44 years had 3.53-fold (aPR = 3.53; 95% CI, 1.57–7.48), 3.55-fold (aPR = 3.55; 95% CI, 1.66–7.60), and 2.70-fold (aPR = 2.70; 95% CI, 1.27–5.74) greater likelihood of having access to HIV/STI testing, respectively, than older adults. Marriage was directly associated with access to HIV/STI testing compared with other marital statuses (aPR = 1.63; 95% CI, 1.36–1.96). Similarly, health insurance coverage was associated with a greater probability of HIV/STI testing (aPR = 1.47; 95% CI, 1.25–1.73). Regarding the duration of residency in Peru since arrival, living for > 1 year in Peru was significantly associated with increased access to HIV/STI testing (aPR = 1.53; 95% CI, 1.09–2.15) compared with residing for fewer months. Other covariates directly associated with access to HIV/STI testing were perceived discrimination (aPR = 1.21; 95% CI, 1.04–1.40) and residing in Chiclayo (aPR = 1.57; 95% CI, 1.24–1.98), Chimbote (aPR = 1.79; 95% CI, 1.41–2.29), and Tumbes (aPR = 1.85; 95% CI, 1.50–2.28). Conversely, being diagnosed with a mental or physical disability (aPR = 0.36; 95% CI, 0.16–0.81) and being employed (aPR = 0.75; 95% CI, 0.65–0.87) were associated with decreased access to HIV/STI testing (Table 4).

**Table 4:** Factors associated with HIV/STI testing among female respondents (*n* = 3,984)

Characteristics		Crude^a^			Adjusted^b^		
		cPR^c^	95% CI^d^	*p*-value	aPR^e^	95% CI^d^	*p*-value
Age (years)							
	18–24	3.67	1.74–7.74	**0.001**	3.53	1.67–7.48	**0.001**
	25–34	4.11	1.95–8.65	**< 0.001**	3.55	1.66–7.60	**0.001**
	35–44	2.94	1.40–6.16	**0.004**	2.70	1.27–5.74	**0.010**
	45–54	1.89	0.86–4.16	0.109	1.91	0.87–4.17	0.105
	55–64	0.93	0.33–2.58	0.892	1.01	0.36–2.81	0.973
	65 or older	Ref.	Ref.	Ref.	Ref.	Ref.	Ref.
Education level							
	No formal education or primary	0.83	0.64–1.09	0.189	1.10	0.85–1.43	0.459
	Secondary	0.86	0.74–1.00	0.056	0.93	0.80–1.09	0.400
	Higher	Ref.	Ref.	Ref.	Ref.	Ref.	Ref.
Mental or physical disability							
	Yes	0.24	0.11–0.56	**0.001**	0.36	0.16–0.81	**0.015**
	No	Ref.	Ref.	Ref.	Ref.	Ref.	Ref.
Employment status							
	Employed	0.79	0.68–0.92	**0.002**	0.75	0.65–0.87	**< 0.001**
	Unemployed	Ref.	Ref.	Ref.	Ref.	Ref.	Ref.
Health insurance							
	Uninsured	Ref.	Ref.	Ref.	Ref.	Ref.	Ref.
	Insured	1.50	1.28–1.77	**< 0.001**	1.47	1.25–1.73	**< 0.001**
Time residing in Peru (in months)							
	0–6	Ref.	Ref.	Ref.	Ref.	Ref.	Ref.
	7–12	1.52	0.98–2.37	0.058	1.48	0.97–2.26	0.068
	More than 12	1.74	1.24–2.44	**0.001**	1.53	1.09–2.15	**0.014**
Marital status							
	Married or living with a partner	2.01	1.68–2.39	**< 0.001**	1.63	1.36–1.96	**< 0.001**
	Other	Ref.	Ref.	Ref.	Ref.	Ref.	Ref.
Perceived discrimination							
	Yes	1.29	1.11–1.50	**0.001**	1.21	1.04–1.40	**0.009**
	No	Ref.	Ref.	Ref.	Ref.	Ref.	Ref.
City of residence							
	Arequipa	1.06	0.81–1.39	0.628	0.99	0.76–1.29	0.966
	Chiclayo	1.48	1.18–1.85	**0.001**	1.57	1.24–1.98	**< 0.001**
	Chimbote	1.85	1.47–2.33	**< 0.001**	1.79	1.41–2.29	**< 0.001**
	Ica	1.25	0.96–1.64	0.094	1.16	0.90–1.49	0.249
	Lima	Ref.	Ref.	Ref.	Ref.	Ref.	Ref.
	Piura	1.08	0.85–1.39	0.495	1.11	0.86–1.43	0.410
	Trujillo	0.97	0.77–1.22	0.822	0.99	0.78–1.25	0.947
	Tumbes	1.84	1.48–2.29	**< 0.001**	1.85	1.50–2.28	**< 0.001**

^a^ Poisson regression

^b^ Poisson regression adjusted per all model variables

^c^ cPR: crude prevalence ratio

^d^ 95% CI: 95% confidence interval

^e^ aPR: adjusted prevalence ratio

## Discussion

This study sought to identify the factors associated to having access to HIV/STI testing among Venezuelan migrants residing in Peru. Only 19.85% and 25.16% of male and female respondents in this study had access to HIV/STI testing. Considering that sex was found to be an effect modifier, all statistical analyses were stratified by sex. Regardless of sex, some variables exhibited a similar direction of the association, including having health insurance, marital status, and city of residence. Nevertheless, specific sociodemographic factors related to a higher probability of access to HIV/STI testing were identified for male and female migrants. These findings may help inform public health policies to promote HIV/STI surveillance in this vulnerable group.

Only 19.85% of male and 25.16% of female Venezuelan migrants and refugees in Peru had access to HIV/STI testing, indicating that less than three out of ten individuals were tested. This finding is inconsistent with those of other studies among Venezuelan migrants living in Trinidad and African migrants living in China, where 41% and 72.9% of migrants, respectively, have been tested for HIV since their arrival [16, 17]. Nevertheless, only the latter study was nationally representative, and both studies investigated only HIV testing. Our findings are consistent with those of previous studies that have highlighted several challenges faced by this population, including difficulty accessing COVID-2019 vaccination [18] and food insecurity [19]. Conversely, it is important to consider the existing conditions for HIV/STI testing among nonmigrants in Peru. In the published literature, we could not identify any studies assessing the performance of HIV/STI testing in the general population in Peru; however, in a study, most men who have sex with men and transgender women indicated that they had undergone HIV testing at some point in their lives, although only 6.2% reported a mean of two tests per year, as recommended by the Peruvian Ministry of Health [20]. Access to HIV/STI testing, treatment, and follow-up is provided free of charge by the Ministry of Health to Peruvian residents and migrants [21].

In this study, health insurance was associated with a greater probability of access to HIV/STI testing among Venezuelan migrants. Previous studies have shown that health insurance coverage in migrant populations increases their use of medical services, including HIV/STI testing, and this phenomenon has also been described in the Venezuelan population [22–24]. In Colombia, the country that has received the highest proportion of Venezuelan migrants ahead of Peru and Chile, studies have documented an increase in the number of Venezuelans seeking medical care despite access barriers owing to the migratory status of this population [25]. Hence, there is a need to develop policies regarding access to STI care in the migrant population in Peru, recognizing that the migrant status itself is a risk factor for these diseases. In Peru, there are some facilities to expedite immigration procedures for people with HIV because of their vulnerability [21]. Regarding the place of residence, HIV/STI testing was more accessible in some regions compared to that in the capital region. This may be attributable to the development of campaigns at the local level, allowing greater access to screening in these locations than in Lima.

Marital or cohabiting status was associated with an increased probability of having undergone a screening test for HIV/STI in both female and male migrants. From a partner protection perspective, immigrants with a previous partner have been shown to exhibit a greater interest in verifying their health status regarding STIs; in addition, couples may undergo this type of test as part of the relationship formalization process and when planning to conceive [26–28]. Couples in a formal relationship are more likely to represent this situation [29]. Considering that several migrants are young and undergo the migratory process as a couple, the reasons described may influence the performance of HIV/STI testing.

Men who identify as LGTBI were more likely to be screened for HIV/STI than heterosexual men. Despite the lack of official data, the Venezuelan LGBTI community is likely to have a higher prevalence of HIV than the heterosexual community, as found in neighboring countries, which would predispose them to more frequent tests [30]. Some barriers that hinder access to HIV/STI testing among the LGBTI community include stigmatization, lower socioeconomic status, distance from screening centers, fear of being diagnosed and the subsequent side effects of antiretroviral drugs, and fear of medical attention and associated expenses [31–33]. Our study calls for an in-depth study of the motivations of Venezuelan migrants in Peru to undergo HIV/STI testing. Likewise, having a secondary educational level compared with a higher one was related to a lower probability of being screened for HIV/STI. In previous studies conducted in Europe and Africa, individuals with higher educational levels were found more likely to undergo screening for infectious diseases, which is explained by their greater awareness of the health risks posed by infectious diseases and the benefits of early diagnosis [34–36]. Future studies should investigate the determinants of HIV/STI testing in these subgroups of migrant males.

In the case of female migrants, those aged < 45 years showed a higher probability of undergoing screening tests. In the general population, younger individuals typically have a higher screening rate than older adults [37, 38]. This may be attributable to greater sexual activity and greater turnover of sexual partners among younger individuals. Consequently, the greater risk of acquiring an STI increases their interest in screening for these infections.

Venezuelan migrants residing in Peru for more than 12 months showed a significantly higher probability of accessing HIV/STI testing. Although a longer residence duration increases the probability of medical attention, including undergoing screening tests to identify or rule out diseases, female migrants are likely to have greater access to HIV/STI testing owing to obstetric or family planning care [39]. In female migrants, perceived discrimination was associated with a higher probability of having access to HIV/STI testing. However, previous studies have identified discrimination against migrants as a barrier to medical care [40–43]. Therefore, further studies are warranted to explain this inconsistent finding.

Having a mental illness or a physical disability was related to lower access to HIV/STI testing in our study. Moreover, being employed was associated with a lower probability of having access to HIV/STI testing. Individuals with mental health conditions and physical disabilities are less likely to undergo these types of screening because of their health condition and prioritization of other types of care [44]. However, high-risk sexual practices have been described in both groups [45, 46]. Hence, concerted efforts are required to promote HIV/STI screening. Employed female Venezuelan migrants were less likely to have access to HIV/STI testing than those who were unemployed. This can be explained by the lack of time to go to screening centers, as more than 90% of the Venezuelan migrant population has informal jobs in Peru. These jobs are characterized by long working hours and low wages [47], thereby creating more barriers to HIV/STI testing.

## Strengths and limitations

Some limitations of this study should be acknowledged. First, this was a secondary analysis of data from a cross-sectional survey, and the study design does not permit to establish causality. Nevertheless, given the variables analyzed in this study, the phenomenon of reverse causality is unlikely. Furthermore, due to differences in the availability of tests for HIV and other STIs and differences in the associated stigma, separate analyses of HIV and STI testing may yield greater insights. Second, responses are liable to be affected by social desirability and recall biases. However, the questionnaires were administered by well-trained pollsters, which may have helped minimize such biases. Third, ENPOVE-2022 is representative only of the eight most populated cities by Venezuelan migrants. Despite these limitations, the use of a representative database with a large sample size was a study strength. Moreover, ENPOVE-2022 was designed by the INEI, a national institution that pioneers population-based health surveys, in conjunction with international institutions that monitor the health status of migrant populations (including the United Nations Refugee Agency, United Nations Population Fund, International Organisation for Migration, United Nations Children’s Fund and World Bank). Therefore, this survey provides robust data. Additionally, because sex was an effect modifier, the analysis was stratified by sex. This approach enabled us to understand the specific factors associated with males and females. To the best of our knowledge, this is the first study to assess access to HIV/STI testing in the Venezuelan migrant population living in Peru.

## Conclusions

Approximately 2 out of 10 Venezuelan migrants and refugees in Peru were screened for HIV/STI, with fewer males than females. Although some factors, such as having health insurance, being married, or cohabiting and living in some cities in Peru, were related to a greater probability of testing in male and female migrants, other variables were only sex-specific. The factors associated with lower access to HIV/STI testing according to sex may help identify subgroups of migrants for targeting screening programs to increase the uptake of HIV/STI screening programs. Developing and implementing sex-specific policies is crucial for increasing access to HIV/STI testing.

## Data Availability

The database of ENPOVE-2022 is freely available on the “Microdatos” website of INEI (https://proyectos.inei.gob.pe/microdatos/). To access the ENPOVE-2022 database, select the survey query tab and choose the “ENPOVE” option, select the year “2022,” and specify the period as “annual.”

## Abbreviations

AIDS: Acquired immunodeficiency syndrome
aPR: adjusted prevalence ratios
ENPOVE: Venezuelan Population Residing in Peru Survey
HIV/STI: Human immunodeficiency virus and sexually transmitted infections
INEI: National Institute of Statistics and Informatics
95% CI: 95% confidence interval
